# Gas Production Strategy of Underground Coal Gasification Based on Multiple Gas Sources

**DOI:** 10.1155/2014/154197

**Published:** 2014-07-09

**Authors:** Duan Tianhong, Wang Zuotang, Zhou Limin, Li Dongdong

**Affiliations:** ^1^School of Mining Technology, China University of Mining and Technology, Jiangsu, Xuzhou 221116, China; ^2^State Key Laboratory of Coal Resources and Mine Safety, China University of Mining and Technology, Jiangsu, Xuzhou 221116, China; ^3^Key Laboratory of Deep Coal Resource Mining, Ministry of Education of China, Jiangsu, Xuzhou 221116, China

## Abstract

To lower stability requirement of gas production in UCG (underground coal gasification), create better space and opportunities of development for UCG, an emerging sunrise industry, in its initial stage, and reduce the emission of blast furnace gas, converter gas, and coke oven gas, this paper, for the first time, puts forward a new mode of utilization of multiple gas sources mainly including ground gasifier gas, UCG gas, blast furnace gas, converter gas, and coke oven gas and the new mode was demonstrated by field tests. According to the field tests, the existing power generation technology can fully adapt to situation of high hydrogen, low calorific value, and gas output fluctuation in the gas production in UCG in multiple-gas-sources power generation; there are large fluctuations and air can serve as a gasifying agent; the gas production of UCG in the mode of both power and methanol based on multiple gas sources has a strict requirement for stability. It was demonstrated by the field tests that the fluctuations in gas production in UCG can be well monitored through a quality control chart method.

## 1. Introduction

1300 m^3^ to 1600 m^3^ of blast furnace gas is generated in the making of one ton of pig iron [1–5]. Forty to fifty percent of the low-calorific-value blast furnace gas is consumed by air heating furnaces of the blast furnaces and most of the remaining, if not properly utilized, cause environmental pollution and energy waste after being emitted to the air. Recovery rate of converter gas (CG) produced in the making of each ton of steel is now 110 m^3^ in advanced countries [6]; however, this figure was 80 m^3^/t only on average among the key iron and steel enterprises in China in 2010. As the second largest product following coke in the coking field, coke oven gas (COG) has an output of 300 m^3^ to 340 m^3^ for each ton of coal, accounting about 15 percent of the coal treated. COG emitted to the air does harm to the environment [3, 4, 7–22]. In 2011, in China, the coke output reached 427.790 million tons and the minimum COG output was at least 170 billion cubic meters, of which about 7 to 9 billion cubic meters was emitted [23]. Thus, in China, a lot of gas is not fully utilized each year, wasting resources, reducing the economic benefits, and polluting the environment.

Conventional utilizing method for COG is producing methanol, methane, or hydrogen. A novel method of producing methanol from COG, involving the CO_2_ reforming of COG to obtain an appropriate syngas for the synthesis of methanol, is proposed by Bermúdez et al. The results suggest that methanol production from COG would be a more attractive alternative to conventional processes [15]. Hydrogen and synthesis gas (syngas) can be produced from steam reforming (SR) of COG. When the reforming gas is used for indirect reduction (IR) of iron oxides in blast furnaces (BFs), carbon dioxide emissions can be lessened [4]. Zhang et al. demonstrated that perovskite-type oxygen-permeable membrane reactors of BaCo_0.7_Fe_0.2_Nb_0.1_O_3-*δ*_(BCFNO) packed with Ni-based catalyst had high oxygen permeability and could be used for syngas production by partial oxidation of methane in COG. Long-term operation test results indicate that the novel tubular BCFNO membrane reactor exhibited not only high activity but also good stability for the partial oxidation of CH_4_ in COG to syngas. Thermodynamic analyses are performed to evaluate the feasibility of H_2_ production from BFG and COG by Chen et al. [3].

In UCG, gas production may fluctuate due to factors such as inrush of ground water, collapse of roofs, and changes of gasifying agent components and gasifier pressures, influencing the subsequent gas utilization technologies [24–31]. In China, the researches on UCG stability are mainly related to high-temperature rock mechanics and the method to control roof collapse through strip skip-mining and filling technologies; however, there are no researches on different requirements of the subsequent gas utilization technologies for the gas production stability of UCG and on the corresponding gas production strategies.

Based on the “Basic Research on the pyrolysis, Gasification and High-temperature Purification of Coal” and “Concept of Poly-generation Industrial Park Based on Coal Cleaning and Coking” of program “973,” Shanxi Key Lab of Coal Science and Technology of TYUT, in 2005, proposed a poly-generation new mode for synthesis gas production based on coal gasification and pyrolysis, that is, a “dual-gas-source” mode. However, there are no documents about the “multiple-gas-sources” utilization mode based on gas sources such as ground gasifier gas, UCG gas, BFG, CG, and COG in China at present.

Based on the industrial ecology theory and the successful experience in “dual-gas-source” poly-generation tests, this paper proposes a “multiple-gas-sources” new utilization mode based on gas sources such as ground gasifier gas, UCG gas, BFG, CG, and COG and, according to the different requirements for the gas production stability of UCG in the two “multiple-gas-sources” utilization modes including power generation, and both power and methanol generation, puts forward strategies and stability monitoring methods for the gas production in UCG. A field test was done. According to result of the field test, gas emission can be reduced; when the gas output or components in UCG fluctuate, output increase or component adjustment of gas can be realized through other gas sources by means of online monitoring of components, pressure, flow, and calorific value of the gas, pressure adjustment of the gas pressurizer, computer solution, and automatic closed-loop control, lowering the stability requirement for the gas production in UCG and creating better space and opportunities of development for emerging sunrise UCG in its initial stage.

## 2. Gas Production Strategies of UCG for Different Ways of Utilization

### 2.1. Gas Production Strategy of UCG for “Multiple-Gas-Sources” Power Generation

In both China and abroad, many power generation tests about the UCG-based gas, BFG, CG, and COG were done and problems such as high hydrogen, low calorific value, and gas output fluctuation were solved, accumulating rich successful experience. As a key restricting project of Magang (Group) Holding Co., Ltd. in the 11th Five-year Plan, the CCPP was completed and put into operation in October 2007. It was the first 150,000 kW combined cycle power plant using gas in China. In relevant test, it succeeded in using both the mixture of BFG and COG and pure BFG. When running with a large load, it can realize quick load reduction at a speed of 78 MW per minute. When running with a full load, its BFG consumption can be reduced by 100 km^3^ in 30 seconds. After that, the steam turbine has a gradually reduced load and, if necessary, runs with the rated speed till disconnection. Upon recovery of gas Lly of the pipe network, it can realize immediate grid connection and on-load run. Practice has proved that the existing gas-steam power generation technology can fully adapt to high hydrogen, low calorific value, and gas output fluctuation. It has been found in multiple facts of UCG power generation; high hydrogen has no impact on UCG power generation technologies. Shandong Energy Xinwen Mining Group Co., Ltd. cooperated with Shengli Oil Field Shengli Power Machinery Group Co., Ltd. in successfully building the first small UCG power plant in China in 2001 step by step. The 12-cylinder 400 kW electrically controlled external combined generating unit highly adapts to the calorific value of underground gas and a hydrogen concentration up to 60 percent as well.

**Figure 1:**
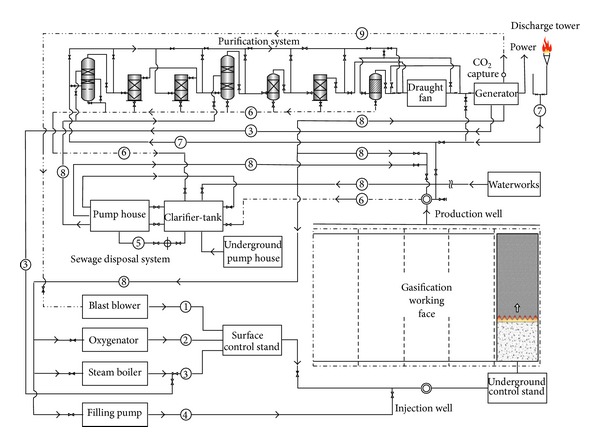
Flow chart of industrial test.

**Figure 2:**
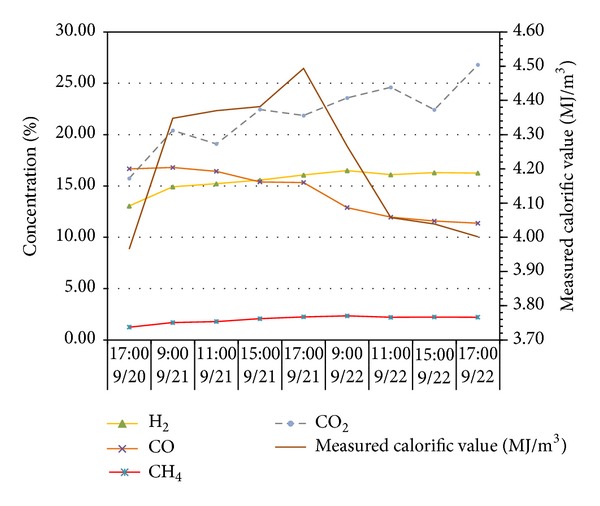
Change of the gas production indices of air gas with time.

**Figure 3:**
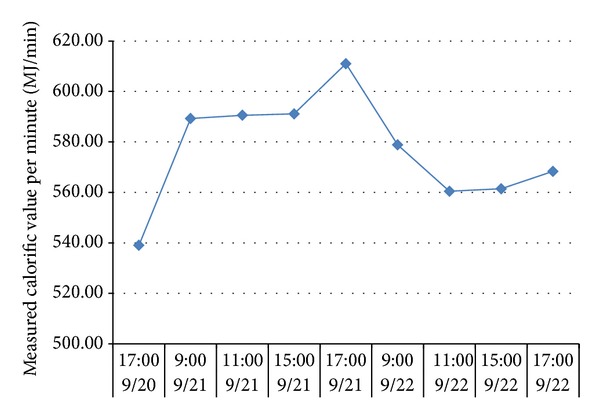
Change of the calorific value of air gas per minute with time.

**Figure 4:**
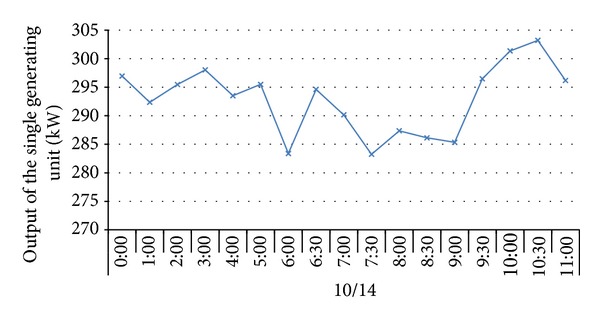
Change of output of the single generating unit after the BFG was used.

**Figure 5:**
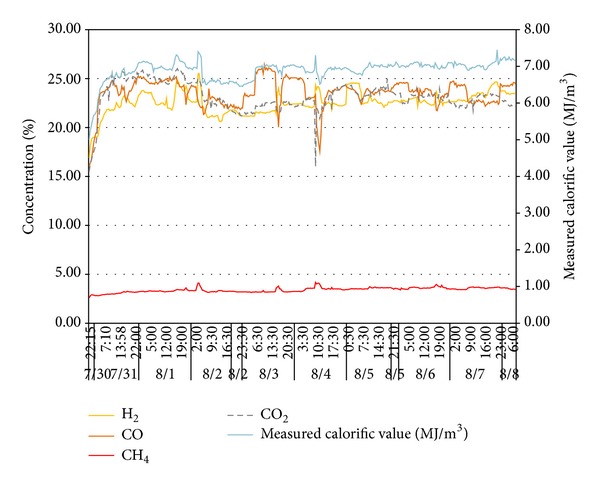
Change of indices of moderately oxygen-enriched steam gas with time.

**Figure 6:**
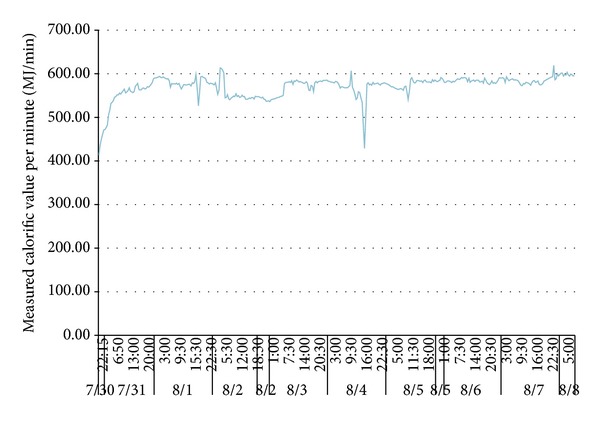
Change of calorific value per minute of moderately oxygen-enriched steam gas with time.

**Figure 7:**
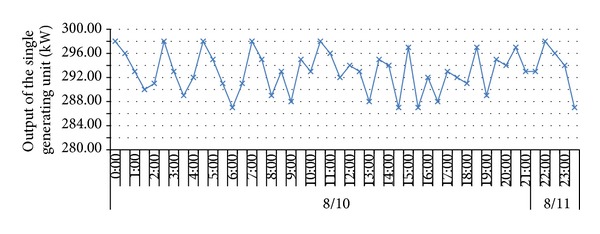
Change of output of the single generating unit after the COG was used.

To verify the impacts of adverse factors such as high hydrogen, low calorific value, and fluctuations of both calorific value and gas output and prove the UCG gas production strategy for “multiple-gas-sources” power generation, the research group with which the author works with did field tests about UCG in a mine. See Figure 1 for process flows of the field tests. In the period, the gases generated by gasifying agents of different concentrations were combined with BFG and COG and used in a power generation test done for a 500GF-PWY generating unit produced by Shengli Oil Field Shengli Power Machinery Group Co., Ltd. From 17:00 of September 20 to 17:00 of September 22 in 2010, the air gas produced in UCG was mixed with BFG and used in another power generation test. In the test, the air gas produced in UCG had a calorific value of 4.10 MJ/Nm^3^ (978.60 kCal/Nm^3^) only on average and fluctuated greatly (Figure 2): the maximum and minimum calorific values were 4.49 MJ/min and 3.97 MJ/min, respectively; the maximum fluctuation was up to 126.27 kCal/Nm^3^; the coefficient of variation was 0.05. In addition, the product of calorific value and flow fluctuated greatly too (Figure 3): the maximum and minimum products were 610.98 MJ/min and 539.02 MJ/min, respectively; the maximum fluctuation was up to 71.95 MJ/min; the coefficient of variation was 0.04. However, after the BFG was used, output of the generating unit became stable and reliable in the whole test; see Figure 4. After the BFG was used, the output of single generating unit fluctuated less than calorific value of air gas per minute as shown in Figure 3. The coefficient of variation of single output of the generating unit was 0.02. This also suggests that air can be used as a gasifying agent. From 22:15 of July 30 to 2:00 of August 8 in 2010, a moderately oxygen-enriched steam gas was mixed with COG and used in another power generation test. In the test, the hydrogen concentration of the gas mixture reached 46.19 percent at most and the generating unit ran stably and reliably, suggesting high adaption of the generating unit to a high hydrogen condition. The moderately oxygen-enriched steam gas generated in UCG had a greatly fluctuating calorific value (Figure 5): the maximum and minimum calorific values were 6.45 MJ/min and 7.45 MJ/min, respectively; the maximum fluctuation was up to 126.27 kCal/Nm^3^; the coefficient of variation was 0.05. In addition, the product of calorific value and flow fluctuated greatly too (Figure 6): the maximum and minimum products were 619.26 MJ/min and 429.07 MJ/min, respectively; the maximum fluctuation was up to 190.20 MJ/min; the coefficient of variation was 0.03. However, after the COG was used, output of the generating unit became stable and reliable in the whole test, as shown in Figure 9. After the COG was used, the output of single generating unit fluctuated less than oxygen-enriched steam gas per minute as shown in Figure 6. The coefficient of variation of the output of single generating unit was 0.01 (Figure 7).

In the power generation test done with a mixture made up of the pure oxygen steam gas obtained in UCG and COG in October 2010, the hydrogen concentration was up to 42.52 percent, suggesting high adaption of the generating unit to a high hydrogen condition too.

In some tests above, steam supply was interrupted due to faults of the steam boiler and the gas produced in UCG had large fluctuations of calorific value and gas output due to factors such as human adjustments of the blower and the oxygen flow; however, after the other gas sources were adjusted, the power generation was stable and reliable. Thus, this paper proposes a gas production strategy of UCG in the mode of “multiple-gas-sources” power generation as below: when the gas produced in UCG serves as a gas source for “multiple-gas-sources” power generation, its output, calorific value, and components may fluctuate greatly because of the adjustment effect of the other gas sources; a high calorific value is not necessary; air can serve as the gasifying agent in the process.

### 2.2. Gas Production Strategy of UCG for “Multiple-Gas-Sources” Power and Methanol Generation

In the industrial ecology, the ecological diversity concept includes the type diversity of the fields and enterprises in industrial parks, scale and contact diversity of enterprises, and diversity of the resource and product contact between the enterprises in industrial parks and those out of industrial parks. Industrial ecology is a systems approach and analogous to natural ecological systems as R. A. Frosch has written: in nature an ecological system operates through a web of connections in which organisms live and consume each other and each other's waste. The system has evolved so that the characteristics of communities of living organisms seems to be that nothing that contains available energy or useful material is lost. Some organisms will evolve that will manage to make their living by dealing with any waste product that provides available energy or useable material. Ecologists talk about a food web: an interconnection of uses of both organisms and their wastes. In the industrial context we may think of this as being the use of products and waste products. The system structure of a natural ecology and the structure of an industrial system or an economic system are extremely similar [6]. Only when the type, scale, and contact diversity of enterprises is maintained can enterprises have stable and efficient outputs. In a “multiple-gas-sources” condition, the enterprises in an industrial park usually cover fields such as UCG, iron making, steel making, coking, IGCC power generation, and methanol production. If there are enterprises engaged in coal bed gas extraction, ground gasification, chloralkali, and synthesis ammonia in an industrial park, the enterprises in the industrial park will have a more obvious type diversity; an enterprise will be able to obtain gas from two or more other enterprises in the industrial park, forming a complicated netlike supply structure with multiple sources of supply. With the supply structure, the enterprises needing gas can obtain gas from the other sources of supply when the gas production of UCG has fluctuated, ensuring a stable supply of gas. This mechanism is similar to the food chain in nature. When the food chain has many species, a kind of species can have more sources of food, and when one of sources of food reduces, they can find other sources of food, thus making the whole food chain and all the species stable.

**Figure 8:**
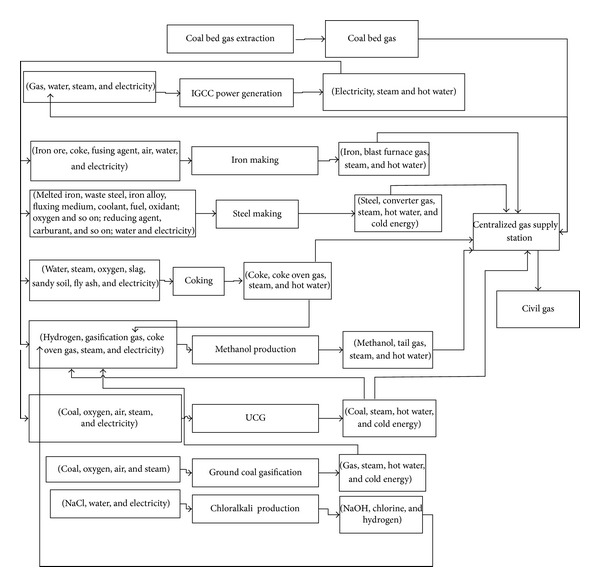
Gas utilization in “multiple-gas-sources” power and methanol generation.

Figure 8 shows the typical gas balance in the mode of power and methanol generation based on multiple gas sources including UCG gas, BFG, CG, and COG. UCG gas, BFG, CG, COG, and methanol-generated tail gas can be provided for an IGCC power plant through a centralized gas supply station. However, BFG and CG are not suitable to serve as raw material gases for methanol synthesizing due to their high nitrogen concentrations while UCG gas and COG are suitable. For UCG gas, deep cooling air separation devices must be used as oxygen generating plants; otherwise, the nitrogen concentration will increase in methanol synthesizing.

When the IGCC power plant is running at full capacity, a part of the gas produced in UCG and COG production is distributed to it; when the IGCC power plant has a power demand smaller than its production capacity, the gas above will not be distributed to the IGCC power plant any longer but to a methanol plant. When the poly-generation system is in a dynamically stable running period, it has a stricter stability requirement for the gas production in UCG than the situation in “multiple-gas-sources” power generation because formula (H_2_ − CO)/(CO + CO_2_) = 2.05~2.15 needs to be met in methanol synthesizing. When the gas production in UCG has fluctuated, adjustment can be done through the ground gasifier gas or the hydrogen produced by chloralkali enterprises, thus reducing the gas production stability in UCG. If there is no ground gasifier gas or chloralkali enterprises, the gas produced in UCG should have stable components and output; otherwise, the components and outputs of the other gas sources will need to be adjusted. Therefore, an effective monitoring method for the components and output of the gas produced in UCG is needed. With the quality control chart method introduced in this paper, abnormal fluctuations of the components, outputs, and calorific values of gases can be found in time.

## 3. Application of the Quality Control Chart Method in Stability Monitoring of the Gas Produced in UCG

As a chart with control limits, the quality control chart in quality management is used for verifying whether quality fluctuations are due to occasional or systematic causes. It can provide information about the existence of systematic causes. On this basis, whether a production process is under control can be judged according to relevant rules of out-of-control or according to relevant rules. There are eight types of quality control charts in all. The specific quality control chart used should be chosen in line with type and quantity of the measured data. In specific application, it is a usual practice to draw three control lines parallel to the horizontal axis of a rectangular plane coordinate system and record the middle solid one that is called center line as CL, with the upper dotted one standing for the upper control limit as UCL and the lower dotted one standing for the lower control limit as LCL. In the $\overset{-}{x}\;$ control chart of the $\overset{-}{x}-S$ control chart,

(1) $$\begin{array}{c} \text{CL}=\overset{-}{\overset{-}{x}},\\ \text{UCL}=\text{CL}+A_{3}\times \overset{-}{S},\\ \text{LCL}=\text{CL}-A_{3}\times \overset{-}{S}. \end{array}$$

In the *S* control chart of the $\overset{-}{x}-S$ control chart,

(2) $$\begin{array}{c} \text{CL}=\overset{-}{S},\\ \text{UCL}=B_{4}\times \overset{-}{S},\\ \text{LCL}=B_{3}\times \overset{-}{S}. \end{array}$$

Coefficients *A*_3_, *B*_4_, and *B*_3_ in formulas (1) to (2) should be chosen according to relevant tables in the quality control chart method.

With the quality control chart method, abnormal fluctuations of the components and outputs of gases can be found and controlled in time. With it, the author successfully developed a program for analyzing continuously monitored data, automatically sending alarm signals, and monitoring the gas production stability in field tests of UCG.

For all links of the process system, the system above the system and the system under the mine both arranged corresponding sensors about temperature, pressure, flow, and components of the gas for real-time monitoring of gas indexes. To make the measured data reliable, the gas flow was measured by a locally displaying flow meter and a sensor that realizes remote transmission of relevant data to the centralized control interface; the gas components were measured by an online monitoring device, a portable gas analyzer, and a chromatograph. The online monitoring periods of flow and components of the gas were 10 minutes and 5 minutes, respectively. In the field tests, real-time control was necessary for fluctuations of components, output, and calorific value of the gas and the main work in the process was real-time adjustments of flow and components of the gasifying agent. Precondition of the adjustments was that abnormalities of components, flow, and calorific value of the gas were found in time.

**Figure 9:**
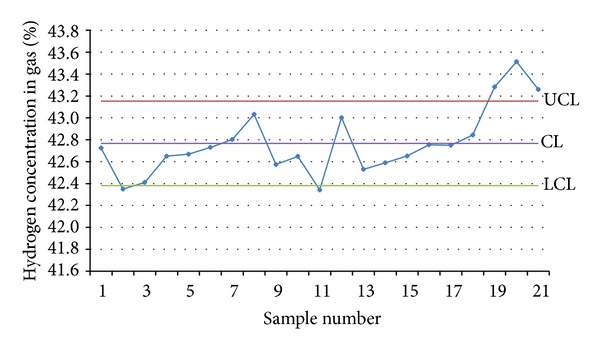
Control chart of average hydrogen concentration in the pure oxygen steam test done on July 4, 2010.

**Figure 10:**
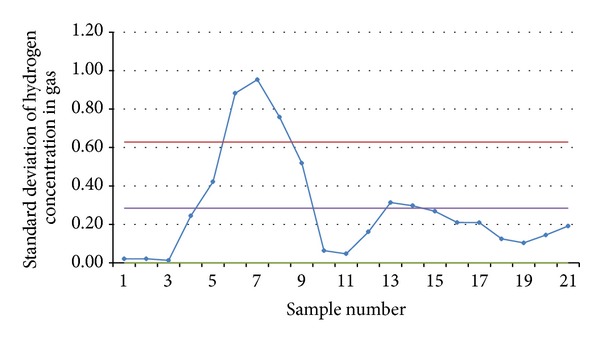
Control chart of standard deviation of hydrogen concentration in the pure oxygen steam test done on July 4, 2010.

In the continuous monitoring in the pure oxygen-steam continuous gasification test done on July 4, 2010, the monitoring program automatically drew an average control chart (Figure 9) and a standard deviation control chart (Figure 10) both about the hydrogen concentration according to the measured data and sounded an alarm to remind the researchers of abnormal fluctuations and of a necessity to find out the causes and make adjustments in time.

Based on the 105 original data obtained in the continuous online monitoring, the monitoring program reached averages and standard deviations of the hydrogen concentration (Table 1). According to the principles for quality control chart choosing, $\overset{-}{x}-S$ quality control charts were chosen. In the control chart of average hydrogen concentration (Figure 9), CL = $\overset{-}{\overset{-}{x}}$ = 42.77; UCL = CL + $1.427\times \overset{-}{S}$ = 43.15; $\text{LCL}=\text{CL}-1.427\times \overset{-}{S}$ = 42.38. In the control chart of standard deviation of hydrogen concentration (Figure 10), CL = $\overset{-}{S}$ = 0.28; UCL = $2.2098\times \overset{-}{S}$ = 0.63; LCL = $0\times \overset{-}{S}$ = 0. In Figure 9, there were three points above the upper control limit, two points below the lower control limit, and seven points showing a trend of continuous increase, suggesting that there are abnormalities. Figure 10 had a similar problem. It was found in comprehensive troubleshooting on the site that the problem happened because the steam boiler providing the underground gasifier with steam failed and started and stopped for several times in the test and, consequently, the gas components changed because of change of the oxygen-steam ratio. After the steam boiler was repaired, both Figures 9 and 10 showed that the gas production was under control. According to results of the field tests, the quality control chart method can realize good monitoring for component, output, and calorific value of the gas produced in UCG.

## 4. Conclusions

1. This paper, for the first time, puts forward a new mode of utilization of multiple gas sources mainly including ground gasifier gas, UCG gas, BFG, CG, and COG. The new mode can not only reduce gas emission, but also, when the gas production in UCG has fluctuated, can get supplementation from the other gas sources, thus lowering stability requirement for the gas production in UCG.
2. In “multiple-gas-sources” power generation, output, calorific value, and components of the gas produced in UCG may have large fluctuations; a high calorific value is not necessary; air can serve as the gasifying agent in the process.
3. The “multiple-gas-sources” power and methanol generation have a stricter stability requirement for UCG than the situation in “multiple-gas-sources” power generation and fluctuations of the gas production in UCG can be well monitored with a quality control chart method.

## Supplementary Material

Surface production system is composed of seven parts, namely, the gasification agent supply system, gasification agent injection drilling, exhaust drilling, purification system, induced draught system, measurement and control system and power generation system.
The second picture shows night view of the field test.
The top part of the third picture shows panoramic view of the field test. The Purification system is shown by the bottom left corner of the third picture and gas injection system is shown by the bottom right corner of the third picture.
The first picture shows different flames of gases generated by gasifying agents of different concentrations in the tests.
The fourth picture shows the power generation system.

## Figures and Tables

**Table 1:** Averages and standard deviations of hydrogen concentration in the gas produced in the pure oxygen steam test done on July 4, 2010.

Number	Hydrogen concentration in gas (%)	Standard deviation of hydrogen concentration
1	42.7	0.02
2	42.4	0.02
3	42.4	0.01
4	42.7	0.24
5	42.7	0.42
6	42.7	0.88
7	42.8	0.95
8	43.0	0.76
9	42.6	0.52
10	42.6	0.06
11	42.3	0.05
12	43.0	0.16
13	42.5	0.31
14	42.6	0.30
15	42.7	0.27
16	42.8	0.21
17	42.8	0.21
18	42.8	0.12
19	43.3	0.10
20	43.5	0.14
21	43.3	0.19
