# Supplementary figures and images for: Gas Production Strategy of Underground Coal Gasification Based on Multiple Gas Sources
Source: ScientificWorldJournal. 2014 Jul 9;2014:154197. doi: 10.1155/2014/154197 (PMC4121015; doi:10.1155/2014/154197)

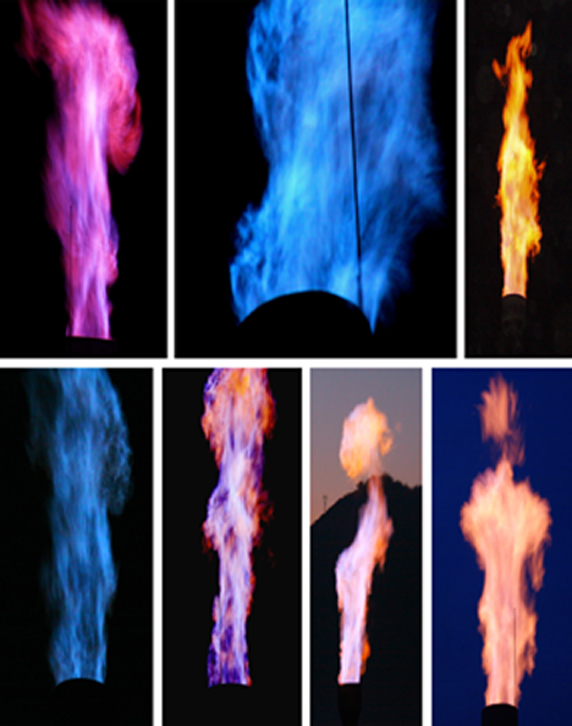

Supplement: Supplementary file 1 — Surface production system is composed of seven parts, namely, the gasification agent supply system, gasification agent injection drilling, exhaust drilling, purification system, induced draught system, measurement and control system and power generation system. The second picture shows night view of the field test. The top part of the third picture shows panoramic view of the field test. The Purification system is shown by the bottom left corner of the third picture and gas injection system is shown by the bottom right corner of the third picture. The first picture shows different flames of gases generated by gasifying agents of different concentrations in the tests. The fourth picture shows the power generation system. [file 154197.f1.zip › 154197.f1/Comparison of torch of different underground coal gasification process .jpg]

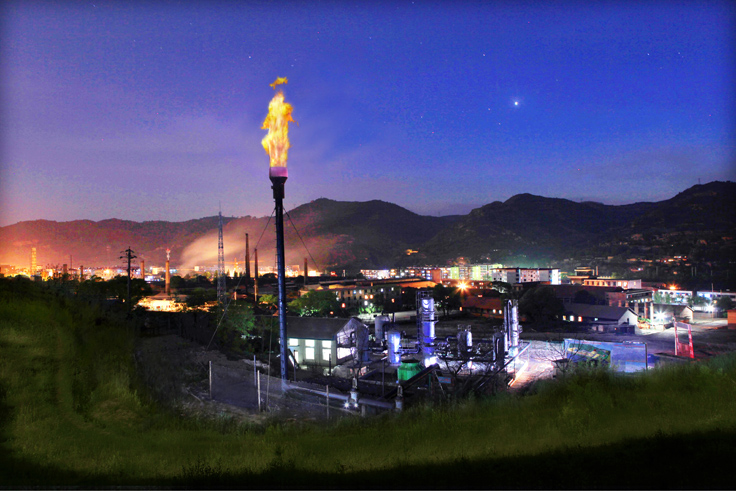

Supplement: Supplementary file 1 — Surface production system is composed of seven parts, namely, the gasification agent supply system, gasification agent injection drilling, exhaust drilling, purification system, induced draught system, measurement and control system and power generation system. The second picture shows night view of the field test. The top part of the third picture shows panoramic view of the field test. The Purification system is shown by the bottom left corner of the third picture and gas injection system is shown by the bottom right corner of the third picture. The first picture shows different flames of gases generated by gasifying agents of different concentrations in the tests. The fourth picture shows the power generation system. [file 154197.f1.zip › 154197.f1/Night view of UCG -IGCC power plant.jpg]

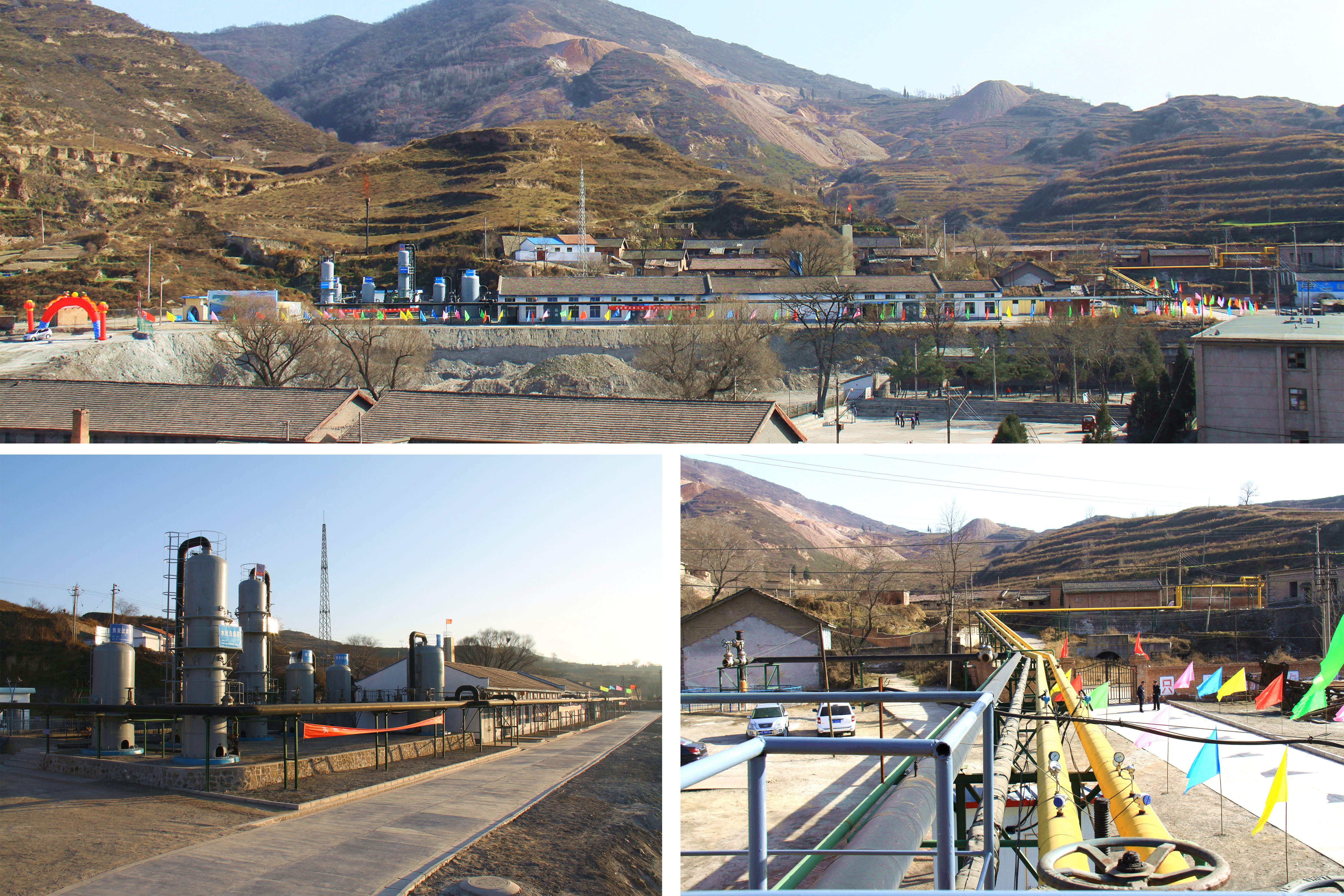

Supplement: Supplementary file 1 — Surface production system is composed of seven parts, namely, the gasification agent supply system, gasification agent injection drilling, exhaust drilling, purification system, induced draught system, measurement and control system and power generation system. The second picture shows night view of the field test. The top part of the third picture shows panoramic view of the field test. The Purification system is shown by the bottom left corner of the third picture and gas injection system is shown by the bottom right corner of the third picture. The first picture shows different flames of gases generated by gasifying agents of different concentrations in the tests. The fourth picture shows the power generation system. [file 154197.f1.zip › 154197.f1/Panoramic view.jpg]

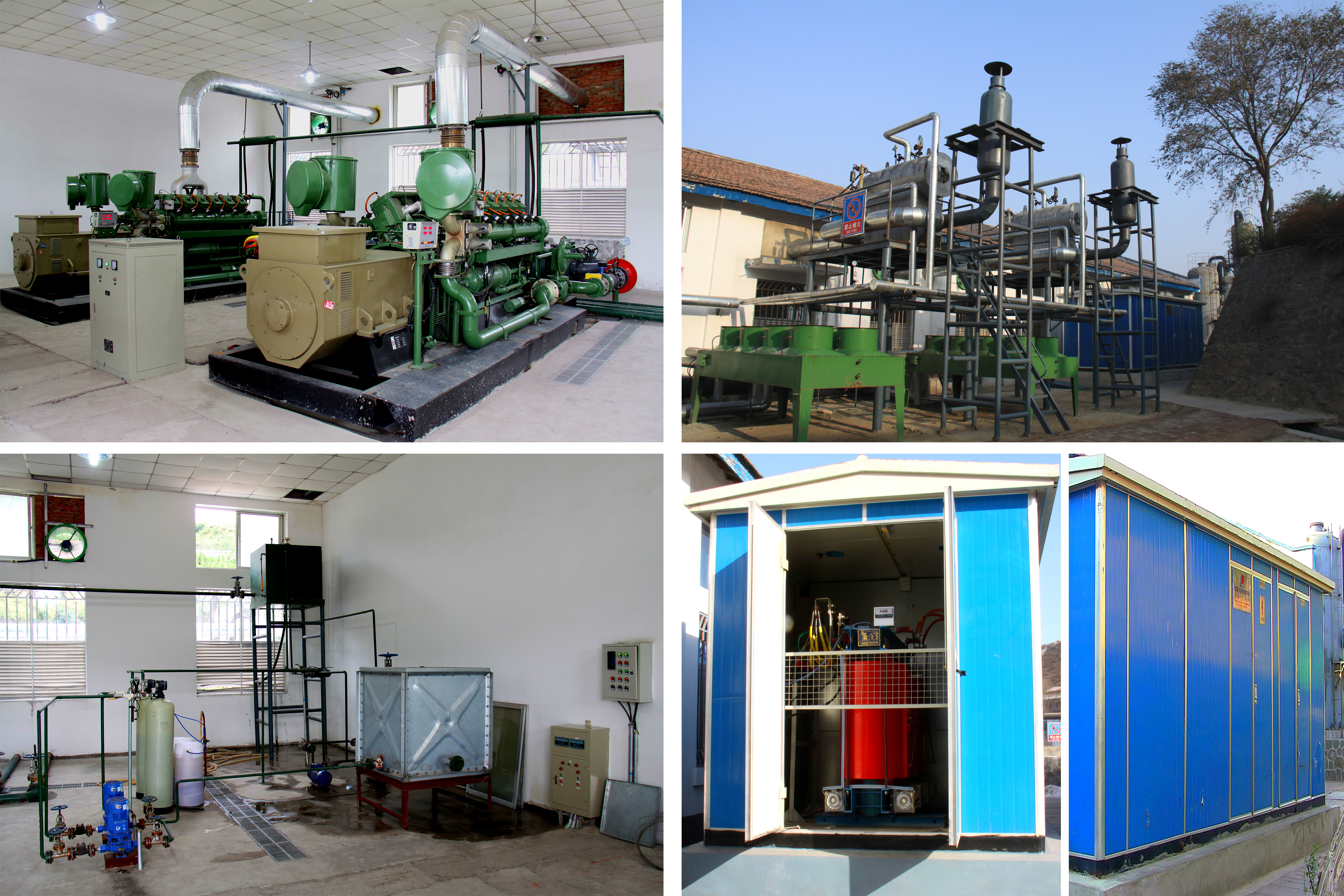

Supplement: Supplementary file 1 — Surface production system is composed of seven parts, namely, the gasification agent supply system, gasification agent injection drilling, exhaust drilling, purification system, induced draught system, measurement and control system and power generation system. The second picture shows night view of the field test. The top part of the third picture shows panoramic view of the field test. The Purification system is shown by the bottom left corner of the third picture and gas injection system is shown by the bottom right corner of the third picture. The first picture shows different flames of gases generated by gasifying agents of different concentrations in the tests. The fourth picture shows the power generation system. [file 154197.f1.zip › 154197.f1/Power generation system.jpg]
